# Clinical Characteristics of 130 Patients With Hepatocellular Carcinoma Followed at a Tertiary Hospital From Brazil

**DOI:** 10.4021/wjon549w

**Published:** 2012-08-26

**Authors:** Andreza Correa Teixeira, Enio David Mente, Cassio Alfred Brattig Cantao, Ajith Kumar Sankarankutty, Fernanda Fernandes Souza, Tatiane Cardoso Motta, Lucas Monsignore, Jorge Elias Junior, Valdair Francisco Muglia, Daniel Giansante Abud, Fernanda Maris Peria, Orlando Castro Silva, Ana de Lourdes Candolo Martinelli

**Affiliations:** a Department of Surgery and Anatomy, Faculty of Medicine of Ribeirao Preto-University of Sao Paulo, Ribeirao Preto, Sao Paulo, Brazil; b Department of Internal Medicine, Faculty of Medicine of Ribeirao Preto-University of Sao Paulo, Ribeirao Preto, Sao Paulo, Brazil; c Department of Pathology, Faculty of Medicine of Ribeirao Preto-University of Sao Paulo, Ribeirao Preto, Sao Paulo, Brazil

**Keywords:** Hepatocellular carcinoma, Liver Cirrhosis, Liver transplantation

## Abstract

**Background:**

Hepatocellular carcinoma (HCC) is a primary malignant tumor of the liver that represents a serious public health problem all over the world, corresponding to the third cause of cancer death worldwide. The object was to present the clinical characteristics and follow-up of patients with HCC attended at the University Hospital of the Faculty of Medicine of Ribeirao Preto-USP (HCFMRP-USP), Ribeirao Preto, Sao Paulo, Brazil.

**Methods:**

Epidemiological and clinical data were revised from medical records.

**Results:**

A total of 130 patients participated in the study, 81.5% of them being males. Mean (± SD) age at the time of HCC diagnosis was 55.6 ± 11.2 years. Cirrhosis was present in 89.2% of cases, with 53.4% of the patients being Child-Pugh A; chronic hepatitis B or C without cirrhosis was detected in 3.2%, non-alcoholic steatohepatitis (NASH) in 3.8%, and a normal liver in 3.8%. Orthotopic liver transplantation was performed in 26.2% of the subjects, 16.9% of the patients were submitted to surgical resection, and 6.2% to percutaneous ethanol infusion (PEI). Transarterial embolization and transarterial chemoembolization were performed in 9.2% of the patients. Systemic chemotherapy was applied to 4.6% of cases and 24.6% of the patients received symptomatic treatment.

**Conclusion:**

Thus, in the present series cirrhosis was the main risk factor for HCC, with 53.4% of the patients being Child-Pugh A. Liver transplantation or surgical resection of the tumor, potentially curative techniques, were possible in only 43.1% of cases.

## Introduction

Hepatocellular carcinoma (HCC) is a primary malignant tumor of the liver that represents a serious public health problem all over the world, corresponding to the fifth most frequent neoplasia among men, the eighth among women and the third cause of cancer death in the world [1, 2]. The rates of HCC incidence are quite variable according to the different geographic regions. According to the data of the International Agency for Cancer Research, 625,000 new cases of HCC per year are estimated to occur in the world, with East Asian and African countries of the Sub-Saharan region contributing with approximately 78% of cases [3]. Seventy to ninety percent of HCC cases occur among patients with cirrhosis or chronic liver disease. Among the main causes of cirrhosis are chronic hepatitis B and C, alcoholic liver disease, and non-alcoholic steatohepatitis (NASH). Hereditary hemochromatosis (HH), α-1 antitrypsin deficiency, Wilson disease and autoimmune hepatites are less frequent causes of cirrhosis [4]. The main risk factor for the development of HCC in the world is chronic hepatitis B virus (HBV) infection, which accounts for 52.3% of all HCC cases [1]. However, the risk factors vary according to different geographic areas and are related to the prevalence of the different etiologies of chronic liver disease and cirrhosis. In Africa and South East Asia, hepatitis B is the main risk factor for HCC, whereas in Japan, Europe and North America, the risk is mainly due to hepatitis C [5].

In the past, HCC was invariably diagnosed during advanced stages of the disease, with few therapeutic possibilities and a rapid fatal outcome. With the advent of new diagnostic techniques and the institution of screening programs for more susceptible individuals, hepatic tumors could be diagnosed earlier and with better possibilities of treatment, including curative ones such as liver transplantation and surgical resection, which have also led to a better understanding of the natural history of this tumor. Today we know that the natural history of HCC consists of three phases: molecular, preclinical, and clinical or symptomatic. The molecular phase includes sequential genetic changes that lead to cell transformation. These changes can be quantitative (loss and gain of chromosome segments), qualitative (point mutations), and epigenetic. The preclinical phase consists of two stages: an initial one during which the tumor is too small to be detected by imaging techniques, and a stage in which the tumor is already detectable (1 to 2 cm in diameter) but still asymptomatic. Finally, there is the clinical or symptomatic phase in which an individual shows symptoms related to the tumor which, in patients with cirrhosis of the liver, normally appear when the tumor reaches 4.5 to 8 cm in diameter [6]. The main symptoms of patients with HCC in the presence of cirrhosis are: hepatomegaly, abdominal pain or discomfort, jaundice, ascites, and constitutional syndrome (weight loss, fatigue, fever and general malaise). In cases in which HCC involves a liver without cirrhosis, a large solitary hepatic mass is usually detected, with infiltrative growth and often vascular invasion and metastasis. The prognosis of HCC is mainly related to the size and number of tumors, the presence of macrovascular invasion and metastases, patient health status or performance status, and the severity of hepatic disease. Tumor progression is the main cause of death in patients with HCC. Other causes are liver failure (7 to 30%), digestive hemorrhage (7 to 10%), infection (2%), and pulmonary embolism (1%) [6]. On this basis, the aim of the present study was to present the clinical characteristics and follow-up of patients with HCC attended at the University Hospital, Faculty of Medicine of Ribeirao Preto-USP (HCFMRP-USP), Ribeirao Preto, Sao Paulo, Brazil.

## Patients and Methods

This was a retrospective cross-sectional study approved by the Research Ethics Committee of HCFMRP-USP (protocol n° 769/2009). A total of 130 consecutive patients attended at the Hepatic Focal Lesions Outpatient Clinic of HCFMRP-USP from 2001 to 2009 participated in the study. Inclusion criteria were individuals of both sexes with a diagnosis of HCC based on the criteria of Barcelona 2000 [7] for the diagnoses performed up to 2006, and on the recommendations of the American Association for the Study of Liver Disease (AASLD) [8] since 2007. Subjects with focal liver lesions differing from HCC, patients who did not fulfill the diagnostic criteria of HCC according to Barcelona 2000 and AASLD recommendations and patients with other concomitant malignant neoplasias were excluded from the study. Clinical and laboratory data were obtained from the medical records of the patients, including age at HCC diagnosis, presence of cirrhosis, etiology of liver disease, Child-Pugh score, MELD (Model for end-stage liver disease) score, BCLC (Barcelona Clinic Liver Cancer) staging classification, tumor type, nodule size, presence of metastasis, presence of portal hypertension syndrome, type of HCC therapy and, alphafetoprotein (AFP) level.

## Results

A total of 130 patients participated in the study, 106 of them (81.5%) males and 24 (18.5%) females. One-hundred-and-sixteen patients (89.2%) were White, eight (6.2%) were Mulattoes, four (3.1%) were Oriental, and two (1.5%) were Black. Mean (± SD) and median age at the time of HCC diagnosis was 55.6 ± 11.2 years and 56 years, respectively (range: 14 - 78 years). Cirrhosis was present in 89.2% (n = 116) of cases, chronic hepatitis B (HBV) or C (HCV), without cirrhosis, in 3.2% (n = 4, two with HBV and two with HCV), NASH in 3.8% (n = 5), and a normal liver was present in 3.8% (n = 5) (Table 1). Among the patients with cirrhosis, the etiology of chronic liver disease was HCV in 35 (30.2%) cases, HCV + alcohol in 29 (25%), alcohol in 23 (19.8%), HBV in 10 (8.6%), cryptogenic in seven (6%), HBV + alcohol in six (5.1%), autoimmune hepatitis in two (1.7%), HH in one case (0.9%), NASH in one (0.9%), HCV + HBV + alcohol in one (0.9%), and HCV + HBV + alcohol + HH in one (0.9%). The two individuals who developed HCC in the presence of chronic HBV infection without cirrhosis presented moderate fibrosis. It was not possible to determine the degree of hepatic fibrosis in the two cases of chronic HCV infection without cirrhosis since one of the two patients was diagnosed with HCC by surgical resection of a bone metastasis, with a later diagnosis of the hepatic lesion by an imaging exam, and the other was submitted to surgical resection of the tumor, although without a free margin or with a biopsy of non-tumoral liver.

**Table 1:** General Characteristics of the 130 Patients With HCC Included in the Study

Characteristics	n	
Sex (male (%):female (%))	130	106 (81.5%):24 (18.5%)
Age at the time of HCC diagnosis (mean ± SD)	130	55.6 ± 11.2 years
Characteristics of the liver (%)	130	
Cirrhosis		116 (89.2%)
Chronic viral hepatites		4 (3.2%)
HBV		2 (1.6%)
HCV		2 (1.6%)
NASH		5 (3.8%)
Normal liver		5 (3.8%)

n: number of patients; HCC: hepatocellular carcinoma; HBV: hepatitis B virus; HCV: hepatitis C virus; NASH: non-alcoholic steatohepatitis.

In the patients with chronic hepatitis C, HCV genotyping was performed in 54/68 cases, with genotype determination being impossible in 14 patients due to the lack of available serum in the Sample Bank and to the fact that the patient had died before the beginning of the study. Thus, among the 54 individuals evaluated, genotype 1 was present in 36 (66.6%), with 11 (20.3%) being subtype 1a, 22 (40.7%) subtype 1b, two of them (3.7%) being subtype 1 and one (1.9%) subtype 1a/1b. Genotype 3 was detected in 18 (33.4%) patients, 15 of them (27.8%) being 3a and three (5.6%) subtype 3. Patients with the remaining genotypes were not identified. In four cases, the diagnosis of HCC was established after sustained virologic response (SVR) to antiviral treatment. DNA quantitation in serum was performed in all 20 cases of patients with chronic hepatitis B. Four (20%) patients presented HBV DNA < 357 IU/mL at the time of HCC diagnosis. These patients had cirrhosis, were HBeAg negative and were taking a nucleoside/nucleotide analogue at the time of HCC diagnosis. Among the individuals with a detectable viral load by the method used in the study, the mean (± SD) value of serum HBV DNA was 1,166,700 ± 2,745,352 IU/mL, with a median of 73,747 IU/mL. The two individuals without cirrhosis who developed HCC had HBV-DNA > 2000 IU/mL. Ninety percent of the patients were HBeAg negative, with only two subjects being HBeAg positive.

Among the 116 individuals with cirrhosis, the Child-Turcotte-Pugh clasification was: A in 53.4% (n = 62), B in 31.9% (n = 37) and C in 14.7% (n = 17). Mean MELD value (± SD), calculated in 111/116 patients, was 13 ± 4, with a range of 6 to 26. Regarding the other two systems of prognostic evaluation used (Okuda and BCLC), 60/115 (52%) patients were Okuda I, 42 (36.5%) Okuda II and 13 (11.3%) Okuda III, and the BCLC results obtained for 114 patients were: BCLC A in 57 (50%) cases (A1: 7 (6.1%); A2: 8 (7.1%); A3: 22 (19.3%) and A4: 20 (17.5%)), B in 22 (19.3%), C in 20 (17.5%) and D in 15 (13.2%) (Table 2).

**Table 2:** Systems for Prognostic Evaluation (Child-Turcotte-Pugh, MELD, Okuda and BCLC) Applied to the Patients With Cirrhosis and Hepatocellular Carcinoma (HCC)

Classification	n	
Child-Turcotte-Pugh	116	
A		62 (53.4%)
B		37 (31.9%)
C		17 (14.7%)
MELD (mean ± SD)	111	13 ± 4
Okuda	115	
I		60 (52.2%)
II		42 (36.5%)
III		13 (11.3%)
BCLC	114	
A		57 (50%)
B		22 (19.3%)
C		20 (17.5%)
D		15 (13.2%)

n: number of patients; MELD: Model for End-Stage Liver Disease; BCLC: Barcelona Cancer Liver Clinic.

Portal hypertension syndrome was detected in 72.3% (94/130) of patients, with portal vein thrombosis identified in 21/128 (16.4%). It was not possible to differentiate between tumoral and non-tumoral portal vein thrombosis. In a screening program of patients with cirrhosis with abdominal US, the hepatic nodule was identified in 59 (50.9%) cases. The diagnosis of HCC was confirmed by two dynamic imaging techniques (three-phase CT, NMR or arteriography) in 54 (41.5%) cases, by one dynamic image in 13 (10%), one dynamic image + alphafetoprotein (AFP) in 28 (21.5%), and by histology in 31 (23.9%), and in four cases (3.1%) HCC was an incidental finding in the explant. Approximately 71% of the patients with HCC had serum AFP < 200 ng/mL. Serum AFP levels < 20 ng/mL were detected in 47.7% of cases, levels between 20 and 200 ng/mL in 23.1%, levels between 201 and 400 ng/mL in 5.4%, and levels > 400 ng/mL in 23.8%.

A uninodular tumor was present in 70.5% (91/129) of cases, a multinodular tumor in 16.3% (21/129) and a diffuse infiltrative tumor in 13.2% (17/129). In one case it was not possible to identify the tumor type. Nodule size was < 5 cm in 59.5% of cases. Tumors between 5 and 10 cm were detected in 19% of patients, and in 8% of cases the lesions were > 10 cm. More than half the cases (55%) fulfilled the Milan criteria. A search for metastases was performed in 117 cases. Four of these (3.4%) had a secondary bone lesion, four (3.4%) had a lesion in the lung, and one (0.9%) patient had an adrenal metastasis (Table 3).

**Table 3:** Characteristics of the Hepatocellular Carcinomas (HCC): Tumor Type, Nodule Size and Evaluation of Metastasis

Characteristics of HCC	n	
Tumor type	129	
Uninodular		91 (70.5%)
Multinodular		21 (16.3%)
Infiltrative diffuse		17 (13.2%)
Nodule size	126	
< 5 cm		75 (59.5%)
5 - 10 cm		24 (19%)
> 10 cm		10 (8%)
Metastasis	117	
Bone		4 (3.4%)
Lung		4 (3.4%)
Adrenal		1 (0.9%)

n: number of patients; HCC: hepatocellular carcinoma.

Histological evaluation of the HCC was possible in 59 cases, with the tumors being classified according to Edmondson-Steiner as: grade I (n = 28 (47.5%)), grade II (n = 11 (18.6%)), grade III (n = 12 (20.3%)) and grade IV (n = 8 (13.6%)). Three patients had the fibrolamellar variant of HCC. Of these, two had NASH and one had a normal liver.

Regarding the treatment instituted, of the 130 HCC patients studied, 50 (38.5%) received an indication of orthotopic liver transplantation (OLT) from a deceased donor. Of these, 34 (26.2%) were submitted to liver transplantation, 12 (9.2%) were on the waiting list for a transplant, and four (3.1%) lost the indication of a transplant while waiting because they no longer fulfilled the Milan criteria. Twenty-two patients (16.9%) were submitted to surgical resection, and eight (6.2%) to percutaneous ethanol infusion (PEI). Transarterial embolization (TAE) and transarterial chemoembolization (TACE) were performed in 12 (9.2%) patients, five of them submitted to TACE, four to TAE, two to TAE + PEI, and one to the combination of TAE + TACE + PEI. Six (4.6%) patients received systemic chemotherapy, one of them with doxorubicin and five with sorafenibe. In 32 (24.6%) cases, symptomatic treatment was the only therapeutic alternative.

## Discussion

In the present study there was a predominance of male patients (81.5%). One of the reasons for the higher HCC rates among males is related to their greater exposure to the different risk factors for chronic liver disease, such as HBV and HCV infection and alcohol consumption [9]. In addition, an experimental study on rats demonstrated that male animals had a two to eight times higher chance to develop HCC, possibly supporting the hypothesis that androgens may influence the progression of HCC and that exposure to risk factors may not be the only feature related to predominance of the disease among males [10]. In the present study, mean age at the time of HCC diagnosis was 55.6 ± 11.2 years. In countries where HBV and HCV infection mainly occurs during adult age, HCC develops less frequently before 50 years of age and the higher incidence rates are observed among individuals older than 75 years [9].

Cirrhosis was present in 89.2% of the individuals with HCC, with HCV infection and alcohol being the main etiologies of chronic liver disease. Epidemiological studies have demonstrated that cirrhosis is the condition most often associated with hepatocarcinogenesis (> 80%), and that the etiology of chronic liver disease is related to the risk factors prevailing in the geographic region studied [11, 12]. In western countries and in Japan, hepatitis C represents the main risk factor for HCC [5]. In Brazil, a recent national survey to update hepatocellular carcinoma epidemiology in the country showed that 98% of the HCC patients (1,279/1,308) had cirrhosis and HCV was the main etiology, followed by HBV and alcohol [13]. According to data of the Health Ministry of Brazil [14], 6,622 new cases of hepatitis C were reported in the Southeast Region in 2008, with 3 560 occurring in the State of Sao Paulo. However, these data are known to be underestimates, mainly because of under-notification. Regarding alcohol as a risk factor for HCC, Donato et al studied 464 individuals with HCC and 824 controls and demonstrated that alcohol ingestion > 60 g/day confers an increased risk of liver tumors and that in individuals with HCV infection the concomitant use of alcohol increases two-fold the risk of developing HCC [15].

Of the 130 patients with HCC, 3.2% had chronic viral hepatitis without cirrhosis, 1.6% of them being infected with HBV and 1.6% with HCV; 3.8% had NASH, and 3.8% had a normal liver. It is well known that chronic HBV hepatitis may be responsible for the development of HCC in the absence of cirrhosis since HBV integration in the genome may determine microdeletions in the DNA of the host and the HBx protein may alter the transcriptional activity by modifying the expression of various genes involved in the control of cell growth [16, 17]. Studies have demonstrated that Caucasians with chronic hepatitis B without cirrhosis and with inactive hepatitis (normal alanine-aminotransferase and low serum HBV-DNA) have a low incidence of HCC [18, 19]. However, additional risk factors should be considered such as advanced age, persistence of viral replication, co-infection with HCV or HIV, as well as the presence of other liver diseases [5]. It is important to point out that the persistence of high HBV-DNA levels (> 10^ 4-5^copies/mL) is a strong risk factor for HCC, regardless of HBeAg, serum aminotransferase levels and presence of cirrhosis [20]. More recently, it has been demonstrated that mutations in the basal core promoter are also an independent factor for the development of HCC [17]. Regarding HCV infection, two individuals without cirrhosis were found to have HCC. In contrast to HBV, HCV has a very low direct oncogenic potential and the main mechanism involved in hepatocarcinogenesis is believed to be represented by the sustained necroinflammatory activity promoted by viral infection, so that practically all cases of HCC in individuals with HCV would occur in livers with cirrhosis or advanced fibrosis. However, several products of the HCV gene (core, NS3, NS4B and NS5A) are known to have the potential for transformation, as demonstrated in murine fibroblast cultures, possibly suggesting that HCV may also have a direct hepatocarcinogenic potential [17]. In a retrospective study of 330 surgically resected HCC, Bralet et al [21] detected 80 cases of non-tumoral livers with minimum or no fibrosis; of these, 30 presented risk factors for HCC, two of them with chronic HCV infection. Over a period of two years, Nash et al [22] detected six cases of HCC in HCV-infected individuals without cirrhosis, one of them with mild fibrosis, four with moderate fibrosis and one with bridging fibrosis. However, additional risk factors were identified in all cases (previous HBV infection in four, excessive alcohol consumption in one, and prolonged immunosuppression in one).

More recently, a group of patients at risk for HCC has been identified, i.e., subjects with SVR after antiviral treatment for chronic hepatitis C. In the present study, the diagnosis of HCC was established after SVR in four cases. Makiyama et al [23] evaluated 3,626 patients with chronic hepatitis C who had received monotherapy treatment with interferon. Of these, 1,197 progressed to SVR, 2.3% of them (27 cases) developing HCC after SVR. When subjects with SVR who progressed or not to HCC were analyzed, most of the HCC cases were found to be males (P = 0.0212), of more advanced age (P = 0.0068), and in a more advanced stage of liver disease before treatment with interferon (P = 0.0345). On the other hand, when HCC patients with and without SVR were compared, sustained responders tended to be older at the beginning of treatment with interferon (P = 0.0552) and at the time of HCC diagnosis (P = 0.0593). Again, males predominated (P = 0.0507). The histological staging and the aminotransferase levels at the beginning of interferon treatment, the time until tumor detection, and tumor size did not differ significantly between the two groups. In another study on 1056 individuals with SVR after treatment for chronic hepatitis C with interferon monotherapy, 29 progressed to HCC. The independent risk factors associated with the development of HCC were: advanced age, high aminotransferase levels and low platelet levels during the period preceding treatment with interferon [24].

In this study, six patients with HCC had NASH, five of them (3.8%) without cirrhosis. In another study conducted in Brazil, Chagas et al [25] detected seven cases of NASH confirmed by histology among 394 patients with HCC (1.7%), all of them negative for other risk factors for chronic liver disease. Of these, six had cirrhosis and one had NASH with only perivenular and pericellular fibrosis. Hashizume et al [26] reported 9 cases of NASH and HCC, six of them in a cirrhotic liver, and three without cirrhosis. Five of the subjects studied were obese, seven were diabetics and eight had dyslipidemia. Similarly, Hashimoto et al [27] studied 348 patients with NASH and no HCC and 34 patients with NASH and HCC, 22% of them having no cirrhosis. Sixty-two percent of the patients were obese, 74% had diabetes mellitus and 29% had dyslipidemia. Kawada et al [28] evaluated 807 patients submitted to hepatic resection of the HCC, 91 of who were negative for HBsAg and anti-HCV. Of the 807 individuals evaluated, eight received a diagnosis of NASH, and seven of these showed no cirrhosis in non-tumoral tissue. The proportion of patients with NASH and HCC without cirrhosis detected in the present study might have been influenced by the method for HCC diagnosis in these cases (histology of the surgical specimen) since surgical resection is one of the main treatments for HCC in a non-cirrhotic liver.

The mechanisms involved in hepatocarcinogenesis in patients with NASH have not been fully elucidated. However, insulin resistance and oxidative stress seem to play an important role in the development of HCC in these patients. Insulin modulates the intracellular signaling through the tyrosine kinase activity of the insulin receptor and therefore defects in this signaling pathway contribute to insulin resistance, which in turn may produce hepatic fat accumulation via lipolysis. The accumulation of fat in the liver with free fatty acids may induce hepatic inflammation by the production of cytokines such as TNF-α. In addition, the insulin resistance associated with NASH inhibits fatty acid oxidation in the hepatic mitochondria, and the intracellular increase of these acids may produce oxidative DNA damage by stimulating microsomal peroxidases. Oxidative stress may also promote carcinogenesis through a product of lipid peroxidation, trans-4-hydroxy -2-nonenal, which may play an important role in the etiology of HCC via mutation in codon 249 of gene *p53* [29, 30].

In the present study, a normal liver was detected in five patients (3.8%) with HCC. In the international literature, 1 to 5% of HCC cases are estimated to occur in a normal liver. HCC in a normal liver is usually detected in young and healthy individuals, with a peak of incidence in the fourth decade of life, with no evidence of chronic HBV or HCV infection or any other type of liver inflammation [31]. Sotiropoulos et al [32] studied 102 non-cirrhotic patients submitted to hepatic resection, 41 of them with an intrahepatic cholangiocarcinoma and 61 with HCC. Approximately 88% of the patients with HCC had tumors > 5 cm, 76.6% of the lesions were solitary, and 24.4% of the tumors were undifferentiated.

In this study, three patients (2.3%) had the fibrolamellar variant of HCC. Of these, two had NASH and one had a normal liver. The incidence of fibrolamellar HCC varies in different geographic regions. In Thailand, a study on 180 individuals with HCC detected a 0.6% rate of the fibrolamellar variant [33]. In Mexico this variant represents 5.8% of all malignant tumors of the liver [34]. The etiology of the fibrolamellar variant of HCC continues to be unknown; however, in contrast to typical HCC, this variant usually occurs in individuals without cirrhosis [35].

Regarding the treatment instituted, 49.3% of the subjects received potentially curative treatment (OLT, surgical resection or PEI). In the case of OLT there was the need for adjuvant therapy as a bridge treatment, with TACE being the main procedure. Brazilian data show that the transarterial procedures are the most common type of initial treatment in HCC patients in our country, with only 4% being submitted to a liver transplantation as an initial therapy [13]. In 2006, the policy of liver allocation in Brazil was switched to the MELD system, with HCC patients having greater opportunities to receive a liver transplant from a deceased donor.

There are some limitations in our results since this is a retrospective study and some information was not available in medical records. However, in a country with a large territory, such as Brazil, marked by social and economic diversity, studies like this are important to show the clinical and epidemiological profile of patients referred to a single University Hospital.

Thus, in the present series, cirrhosis was the main risk factor for HCC, with 53.4% of the patients being Child-Pugh A. A liver transplant or surgical resection of the tumor was possible in only 43.1% of cases. The encouragement of screening programs for the detection of early tumors, efforts to increase organ donation and the evaluation of new diagnostic and therapeutic strategies, including biomarkers, will contribute to a better prognosis for patients with HCC.
